# Is the gap between micro- and macroeconomic assessments in health care well understood? The case of vaccination and potential remedies

**DOI:** 10.3402/jmahp.v2.23897

**Published:** 2014-04-10

**Authors:** Nikolaos Kotsopoulos, Mark P. Connolly

**Affiliations:** 1 Unit of PharmacoEpidemiology and PharmacoEconomics, University of Groningen, Groningen, Netherlands; 2 Global Market Access Solutions, Mooresville, NC, USA

**Keywords:** vaccines, microeconomic, macroeconomic, economic evaluation, multiplier, general equilibrium models

## Abstract

Vaccination is an established intervention that reduces the burden and prevents the spread of infectious diseases. Investing in vaccination is known to offer a wide range of economic and intangible benefits that can potentiate gains for the individual and for society. The discipline of economics provides us with microeconomic and macroeconomic methods for evaluating the economic gains attributed to health status changes. However, the observed gap between micro and macro estimates attributed to health presents challenges to our understanding of health-related productivity changes and, consequently, economic benefits. The gap suggests that the manner in which health-related productive output is quantified in microeconomic models might not adequately reflect the broader economic benefit. We propose that there is a transitional domain that links the micro- and macroeconomic improvement attributed to health status changes. Currently available economic evaluation methods typically omit these consequences, however; they may be adjusted to integrate these transitional consequences. In practical terms, this may give rise to multipliers to apply toward indirect costs to account for the broader macroeconomic benefits linked to changes in health status. In addition, it is possible to consider that different medical conditions and health care interventions may pose different multiplying effects, suggesting that the manner in which resources are allocated within health services gives rise to variation in the amount of the micro–macro gap. An interesting way to move forward in integrating the micro- and macro-level assessment might be by integrating computable general equilibrium (CGE) models as part of the evaluation framework, as was recently performed for pandemic flu and malaria vaccination.

The economic benefit of a healthy workforce on improved productivity has been supported by a range of microlevel evaluations observed in both developed and developing countries 1–4. Instinctively, it is understood that if people are healthy, their capacity to deliver labor to the market is enhanced and absenteeism avoided. Similar conclusions can be drawn for preventative interventions that avoid illness all together. These microeconomic evaluations have been presented in both the developed and the developing world 1–4. When looking at macroeconomic assessment, the evidence has shown that country-specific economic growth rates contribute to better health and increased life expectancy. This relationship occurs through a number of channels, including improved access to health care, more nutritious food, improved sanitation and living conditions, and the relationship between good health and income that is likely bidirectional (5, 6).

Building on the above reasoning, we observe that vaccination is one of the most powerful tools to prevent the spread of infectious diseases in a community. Therefore, investing in vaccination can offer a range of economic benefits of which some are immediate, whereas others may take generations to materialize. However, the benefit of reducing health care expenditure attributed to infectious diseases is perhaps the most frequently described one where vaccines have repeatedly been shown to generate significant health care savings (7). These health cost savings are the focus of most microeconomic evaluations. Meanwhile, when focusing solely on health costs, one may overlook the broader benefits gained, which may not be well captured in microeconomic evaluation frameworks. The benefit of vaccines should be amplified because much more is achieved through the improved health outcomes that, in turn, result in changes in behavior and human capital accumulation hence, giving rise to benefit to be observed at the higher, macro level. The best examples for that are the pre-pandemic flu vaccination programs that impact on short-term and micro- and macroeconomic parameters (8). There have been attempts to establish the long-term impact of pandemic on the economic growth of a country, through the decline in high school graduation, decreased wages, and the increased likelihood of poverty. These assessments used the 1918 pandemic as a basis (9). They later affect the individual but also accrue for a significant time post a pandemic, affecting the economy at a pronounced level (10). Further evidence of the broader vaccine benefits related to improved health condition is vaccines likely to influence education attainment, which can lead to greater lifetime earnings (11). Thus, for vaccines there is evidence suggesting that analysis should go beyond the simple evaluation of microeconomic findings in order to highlight the total benefit.

Economic assessments offer several micro- and macro-evaluation tools that can be used to investigate the benefits. However, most published economic evaluations of vaccines to date have been microeconomic, focusing solely on cost-effectiveness. Fewer economic assessments project the macroeconomic benefits of vaccinations. Both levels of economic assessment are useful for decision making and for resource allocation decisions; however, they may lead to different results with different consequences, and we will expand more on these later in this paper. At the micro level, it is expected that economic assessments will inform the price setting of vaccines. At the macro level, they may inform the prioritization of competing investment initiatives that are deemed as advantageous for the economy.

Surprisingly, there is a known discrepancy in the output reported between micro- and macroeconomic evaluations when the analysis is performed separately. This was recently described in a published review assessing the impact of malaria. Malaney et al. (12) reported that the macroeconomic estimates for the impact of malaria on the gross domestic product (GDP) ranged from 0.25 to 1.3%, whereas the projection of the microeconomic studies to the overall economy suggested a lower economic impact (the GDP impact ranged from 0.14 to 0.62%). The difference between the two evaluation methods does not seem large, but a 1% difference in GDP attributed to one disease that can be prevented is meaningful. An explanation for the difference between micro and macro estimates could be attributed to less well-defined broader benefits that can lead to economic consequences at the level of microeconomic assessment that are often omitted.

This paper advocates for macroeconomic modeling alongside microeconomic approaches. The benefit of analyzing both perspectives is that a combined assessment may more pragmatically quantify the broader economic consequences of investing in vaccines. The combined micro and macro data can then allow decision makers to allocate resources to achieve health and economic priorities. The scope of macroeconomic evaluation is different from ‘value for money’ microeconomic assessments that are widely used in therapeutic drugs. In vaccines, the focus is to capture in addition the rate of return of public health investment (13, 14). Thus, microeconomic methods alone may not be sufficient from a policy analysis perspective. We hereby describe and critically appraise current microeconomic and macroeconomic assessment methods in terms of the ability to encapsulate the broader benefit of prevention and its relevance for decision makers. Moreover, we define the ‘transitional’ economic consequences that may result from preventing communicable diseases between the micro and macro level. In addition, this paper analyzes the differences observed between micro and macro assessments. This identified void should motivate further methodological research. It is likely that an intermediate or ‘transitional’ or ‘meso-economic’ domain could be thought of as a gateway by which the micro and macro economy of health are connected. This domain may provide an explanation of the differences observed between micro- and macroeconomic outcome results.

## Quantitative micro- and macroeconomic analytical methods

### Microeconomic analysis

Microeconomic analysis captures the cost of a communicable disease and the value of prevention for the individual at a community level. The starting point for this analysis is the measurement of the disease burden. These methods quantify the natural history of the disease (frequency in function of demography), disease management cost, and quality-of-life impact. The disease burden estimates are part of a cost-effectiveness or cost–utility analysis that compares alternative health care interventions. The analyses address estimates of incremental cost and outcome of the different interventions considered, such as vaccination compared to no vaccination. The costing is typically based on a prospective algorithmic simulation of short-term natural history and treatment pathways of a disease (15). Outcomes may be measured in terms of cases prevented, survival gained (life years gained), quality-adjusted life years gained (QALYs gained), or disability-adjusted life years gained (DALYs gained) from the intervention. Although quality-of-life self-reported assessments allow the inclusion of preferences, this short-term and simplified microeconomic framework would not quantify broader consequences such as changes in economic behaviors, school attendance, and school attainment. It is only through longer term macroeconomic observation that the discrepancy can be observed, giving rise to varied microeconomic and macroeconomic observations.

For many communicable diseases in the developed and developing world, the indirect costs of lost productivity will exceed the medical cost of a disease (16, 17). Hence, additional methods should be considered to capture the full benefit of vaccination. Loss of productivity is quantified using either the human capital or the friction cost method. The former assumes that the foregone output could be plausibly captured by the marginal product associated with a worker (summed over the period of illness, or the worker's remaining time in the workforce). In practical terms, the loss in productivity cost for an illness calculated by the human capital method is the worker's wage (the measure of his or her productivity) multiplied by the length of his or her absence from work (18). The friction cost method estimates its cost by using the worker's wage multiplied by the minimum length of absence and the average friction period in the economy (i.e. the average duration of a vacancy until their labor has been replaced) (19). The underlying assumption is that the output lost is due to the friction in the labor market that restricts the ability of employers to find a substitute for the sick worker, but in addition, the losses are limited to the time that is required for the worker to be replaced. Both the human capital and friction cost methods provide a monetary measurement either from the perspective of the individual or from the perspective of the firm, respectively (20). The resulting monetary benefit may be compared with the cost of vaccination in a cost–benefit analysis, which typically generates estimates of the Net Present Value or the Internal Rate of Return.

Valuation of absenteeism requires evidence on the impact that a disease has on workdays lost, but a next stage of higher precision on productivity assessment is the quantification of *presenteeism*. This is even more difficult to assess correctly than absenteeism, since data on the productivity of disease-impacted workers while at work are scarcely available and prospective data collection is needed. Nevertheless, survey methods, using metrics of productivity while at work, have been developed (21) and could be employed to quantify the productivity effect aside from the mere measurement of absence from work. Thus, some specific methods may be able to capture a fragment of significant broader consequences. In addition, through econometric methods the broader consequences of educational attainment and school attendance may be integrated to assess the benefits of preventing a communicable disease on individuals’ lifetime earnings.

Lastly, another method originating from the theory of microeconomics that evaluates the overall value of a new intervention within a broader context is the ‘contingent valuation method’. According to this survey method, respondents are asked to evaluate a question and place a value upon a hypothetical market product described (22). The ‘contingent valuation method’ collects preference information from the respondents by asking them how willing they would be to pay for the provision of a health care service or how willing they are to pay in order to avoid a disease. The results of this survey method are expressed in monetary terms, and hence they can be used in a cost–benefit analysis of a vaccination. Depending on the design of the survey, ‘contingent evaluation methods’ may provide estimates of the value that the society attributes to the reduction of risk of infection after taking into account an array of intangible benefits (e.g., ability to save money, food security, risk of catastrophic expenditure, and fertility). The ‘contingent valuation method’ reveals individuals’ preferences and valuation, which, in turn, implicitly takes into account several evaluations that individuals consider prior to deciding on their economic and social behaviors.

The intangible broader benefits of health would be immediately recognizable to individuals because these attributes reflect the way that people live. As such, contingent valuation may be able to quantify a range of broader consequences as attributes with corresponding monetary values assigned to these benefits. The comprehensive nature of the method suggests that individuals factor in a range of parameters likely to be relevant to macroeconomic analysis. Hence, contingent valuation estimates are greater than disease burden estimates due to the interpretation of these future benefits that would not be captured using other microeconomic analysis methods.

### Macroeconomic analysis

Macroeconomic analytical methods typically apply retrospective cross-country time series data to assess the impact of a disease on the GDP per capita after controlling for confounding factors. They can follow an assessment across different economic sectors, including health care, but at the country level. Such models have been used to assess the macroeconomic impact of malaria, tuberculosis, and HIV to estimate the impact of disease on GDP (23). Importantly, they do not always define the precise mechanism by which a communicable disease influences economic growth. In addition, econometric analyses combine aggregate secondary macroeconomic and epidemiological data. Macroeconomic models may omit, due to the absence of detailed historical data, critical variables such as access to health care per country, health care infrastructure, or adoption of health technology per country. Moreover, the estimation and isolation of the benefits of vaccinations on the GDP require sufficient retrospective data from countries with vaccination programs. The collection of prospective macroeconomic and epidemiological data with the view of assessing the relationship between prevention of a communicable disease and the macro economy could be very time-consuming and resource intensive. Nevertheless, these methods may inform robust cross-country economic analyses of vaccination benefits.

The availability of GDP estimates linked to health investment and health status changes and compared with microeconomic estimates might help to estimate economic multipliers. For instance, dramatically reducing malaria disease in a region may improve the economy overall of the region, as parents may better care for their children to give them a better education. It is conceivable that should these multipliers be identified, they could be applied in microeconomic models as well to account for underestimates obtained. The identification of multipliers is already established in fiscal policy, recognizing that public investments can lead to greater returns than the actual fiscal investment. In this context, one can envisage that multipliers could be applied to health to reflect broader consequences of health gains in microeconomic analysis (24).

## The added value of macroeconomic assessment

The mechanism by which a disease influences the economy is multifactorial, with large economic externalities likely associated with the prevention of highly burdensome diseases (25, 26). A recent systematic review of studies evaluating the broader economic impact of vaccination in low- and middle-income countries identified a diversified set of categories (27). They ranged from cost savings and increased productivity to ecological externalities, influencing fertility rates, and longer term economic sustainability. The use of broader economic benefit variables for vaccines implies that there is not always a perfect match between the results of micro- and macroeconomic assessment.

Undoubtedly, the most dramatic consequence of infectious diseases is mortality. In addition to quantifying the cost of dying (e.g., terminal care and funeral costs), microeconomic methods have measured the mortality impact on foregone lifetime earnings. Meanwhile, increased mortality may have broader consequences. The impact of vaccination on mortality may be regarded as a microeconomic benefit first, which can be quantified. It may subsequently have an impact on the change in fertility rates that influences the demographic composition and may be deemed as a broader consequence, for which quantification is more complex and may take considerable time (generations) to illustrate the impact (28). Endemic malaria affects the demographic structure of a society, as the mortality burden falls mainly on infants and children under the age of 5. Young-age mortality as a result of malaria and/or HIV/AIDS infection reduces the macroeconomic prospects of affected countries (17). There are similar scenarios as in the recent SARS outbreak causing a high mortality rate, but where the greatest impact felt was not in the health care sector but in tourism and travel sectors, which would affect the GDP directly (29).

Moreover, the persistence and high prevalence of communicable diseases are likely to exacerbate the economic impact by altering the social and economic behavior of individuals. At the individual level, when a communicable disease is circulating in the community, the perception of infection's risk will likely influence short- and long-term economic decision making (17). Hence, in countries where HIV/AIDS is highly prevalent, individuals may consume and invest less in order to protect themselves against the possibility of disease-related catastrophic expenditure (17). Altered consuming and investing decisions may negatively affect the sustainability of the economy as they reduce capital formation by discouraging individuals from saving. In addition, capital shortages and reduced consuming may discourage foreign investors.

The fear of infection may also impact mobility, which may have a cross-sectorial impact that limits trade. Furthermore, the affected households may divert productive resources to caring, and the high prevalence of endemic diseases diverts public resources to health care and thus limits the funds for investment in other sectors. Furthermore, communicable diseases such as malaria may interfere with long-term cognitive development (30). The long-term neurological sequel of malaria, the presence of anemia, but also the uncomplicated malaria episodes are thought to impact school attendance, educational attainment, drop-out rates, and overall cognitive development. These consequences are seldom quantified in economic terms (31).

In human capital economics, the most explored causal relationship is between earnings and the quantity and/or level of education attained (32–35). Reduced school performance has some immediate societal cost implications, but, more importantly, it may reduce lifetime opportunities because of education-linked wage effects that will persist over the course of life 36–38. It translates to lower lifetime earnings that may result in less tax revenues and thus fewer resources available for public investments that could vitalize the economy (38). In addition, reducing a country's human capital accumulation may undermine an economy's long-term prospects, its competitiveness, and its attractiveness to foreign investors.

A prominent and well-studied economic parameter relates to labor productivity. A highly endemic communicable disease may result in sick days (absenteeism). Sick days pose a burden to the individuals as they may represent a considerable proportion of an individual's earnings. In addition, sick days result in substantial loss for the firm, which reduces its profitability. Furthermore, reduced productivity while at work (presenteeism) may pose a substantial economic burden on firms due to the productive output foregone. Although the friction cost method suggests the work can be made up or performed by colleagues, the absolute output reduced can be extrapolated. Projecting productivity to a group of firms that operate within an affected area constitutes a perfect example of a broader consequence that, in the long run, undermines macroeconomic growth through the reduction of total productivity and foreign investments. These elements are neglected in microeconomic analysis.

**Fig. 1:**
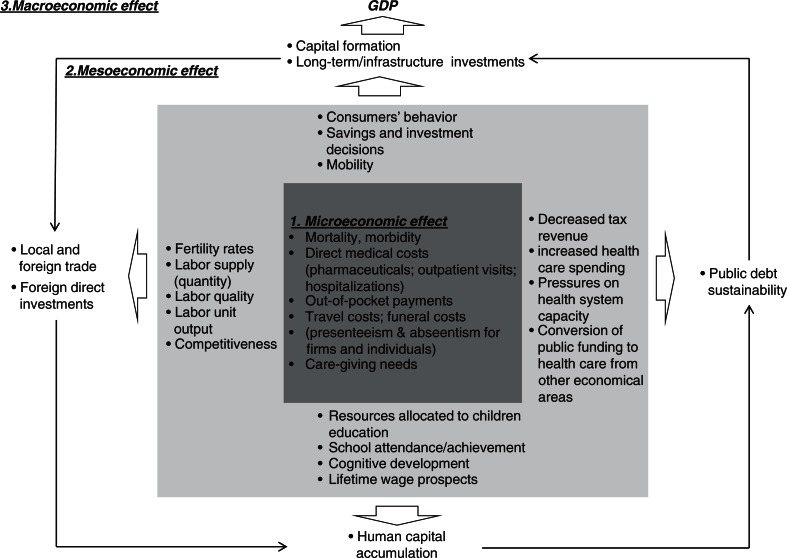
Communicable diseases and the economy.

Figure 1 illustrates the mechanisms with which a communicable disease may influence the broader economy. The multidimensional relationship between the economy and communicable diseases suggests that a preventative measure such as vaccination may benefit the economy in multiple ways and that benefits transcend the microeconomic value to individuals and households. An array of broader benefit appears to intermediately affect the economy and magnify the microeconomic economic consequences of a communicable disease and therefore the benefit of prevention. The critical broader benefit that originates from the prevention of highly burdensome communicable diseases may have large macroeconomic consequences that need to be considered quantitatively or qualitatively in the analysis and policy decision-making process. Quantifying the microeconomic benefit appears to be relatively straightforward, however; microeconomic consequences appear to be isolated to those that are immediately measurable and a small fraction of the potential macroeconomic benefit. In what follows, a short description of the current economic evaluation methods is presented, and the extent to which they can integrate the quantification of broader benefit is discussed.

## Filling the micro–macro void

Vaccinations offer far-reaching economic benefits in both short- and long-term periods. Economic theory provides microeconomic and macroeconomic methods for evaluating the benefits of vaccinations. They are often first projected using microeconomic methods that demonstrate how vaccines can generate productivity gains through reduced morbidity and mortality. Establishing the causality between vaccines at the macroeconomic level is more difficult to obtain due to the societal transformations that vaccines can provide. Vaccines may result in a cluster of transitional or broader consequences that may have a considerable impact on the macro-economy. They not only save lives but also change behavior and investment choices domestically and internationally. For example, investigations have noted differences in GDP per capita between countries based on the presence or absence of malaria (23). However, causality at the macro level requires knowledge about the far-reaching aspects of societal change that will occur in order to make macro-level predictions. In addition, a population-based vaccination may improve the quality and patterns of care provided within a country by preventing a substantial number of cases in need of secondary or tertiary care. Thus, although vaccination may increase the quality, efficiency, and productivity of a health care system, the benefit of a population-based vaccination may take several years to materialize, and prospective studies are needed to generate the corresponding evidence.

Microeconomic and macroeconomic analyses offer useful policy-making tools. Microeconomic analyses may inform budget-efficient decisions and inform scarce resource allocation decisions within the health care system. Absenteeism quantification methods quantify the value of productivity loss, which represents the most considerable cost component in developing countries. Contingent valuation methods are well established theoretically and may be valuable for countries with high out-of-pocket payments. The results of macroeconomic analyses are presented in terms of GDP thus; they allow the comparison of vaccinations with other interventions across different sectors (e.g., transportation, environment, and education). Macroeconomic analyses may offer a tool for cross-sectorial or general government (or foreign aid) efficient budget allocation of resources. Macroeconomic analyses may be used to benchmark countries and identify best practices and thus inform future policy that could be internationally implemented. Although of limited value to health care budget holders, macroeconomic analyses may be particularly relevant for international organizations and general governments.

The broader consequences of vaccinations may resemble what Schumpeter described as ‘meso-economic’, which constitutes *a structure component of a ‘deep’ invisible macro structure* (39). The concept of the meso-economy is a debatable construct of which a thorough analysis is beyond the scope of this paper. What this paper advocates is that this domain could also be thought of as a gateway by which the micro and macro economy of health are joined (see Figure 1). Currently available economic evaluation methods typically omit these consequences, however; they may be adjusted to integrate some of the ‘meso-economic’ consequences.

The observed gap between micro and macro estimates in health presents challenges to our understanding of health-related economic benefits and how one might address the gap methodologically. Foremost, the micro–macro gap suggests that microeconomic methods applied in health do not adequately reflect the broader economic benefits that can be achieved through health-status gains. The underestimates provided by current microeconomic methods would likely vary as the broader consequences would vary for different health care investments. For example, the broader consequences associated with pediatric immunization programs, prostate cancer screening, and cancer interventions would all be different, therefore influencing the micro–macro gap differently.

In the previous section, we proposed the concept of multipliers that could be derived to better understand the relationship between micro and macro health models. Because the externalities attributed to health vary at different stages of life and nature of illness, this would suggest that multipliers applied to health investments would also vary depending on the nature of the condition and age of the individual. In this sense, one can imagine potential weights for different medical conditions that could be applied to microeconomic estimates to understand the macroeconomic consequences of specific interventions.

Another promising way to assess the link between micro- and macro-economic levels related to health investment is the use of model-based analytic methods that have been recently developed to assess the impact of vaccination against communicable diseases, the computable general equilibrium models (CGE) (8). CGE models usually depend on Social Accounting Matrices (SAM) for national income and input–output data. The underlying assumptions of CGE models are that evidence regarding a cluster of broader consequences (as described in Figure 1) can be modeled to simulate the impact of a disease on the macro-economy. CGE models factor in institutional sectors of the economy, including government, households, the main economic sectors (e.g., manufacturing, agriculture, banking, tourism and transport, etc.), capital, and labor as well as foreign trade. CGE methods have been used to assess the impact of pandemic influenza on the UK economy (8). It was modeled in terms of reducing the quantity and quality of labor supply. They are the instruments that assess the details at micro level, but evaluate the overall benefit and cost at macro level. Those models assumed that labor supply would be affected either through high mortality or through absenteeism and reduced productivity at work (presenteeism).

CGE models have been extensively used for years to inform development policies across several countries (40). A recent study assessed, using a dynamic CGE model, the impact of malaria on the economy of Ghana (41). The study assessed the impact on the GDP per capita as well as the income-distributional effect of malaria-preventing strategies by taking into account labor size and productivity (presenteeism and absenteeism). Current, CGE models heavily rely on assumptions regarding the consequence of a communicable disease on economic behaviors. For CGE models to generate pragmatic estimates of the macroeconomic impact, collecting and collating survey evidences, shedding light to the impact of communicable diseases on a variety of economic behaviors, are needed by constructing a valid analytical framework for the overall economy. Nevertheless, they can provide a comprehensive quantification of the overall economic impact of a vaccination by modeling a variety of broader consequences that can help to fill the micro–macro gap.

## Conclusion

There are reasons to believe that, for high-burden communicable diseases able to spread across an entire community, a full economic package encompassing microeconomic and macroeconomic analyses about new interventions such as vaccines is warranted to inform resource allocation decisions. Each type of analysis may not come to precisely the same end-result about cost-effectiveness evaluation when done separately (see Table 1). Meanwhile, there are methods and techniques now available that could facilitate the build-up of a more direct link between the two levels of assessments such as CGE. It should also be further explored if meso-economics as a new domain of existing in economics could help explaining the discrepancies observed in the results between micro and macro level.

**Table 1:** Micro- and macroeconomic methods for evaluating vaccine indirect costs

	Microeconomic methods				
			
Characteristics	Cost of illness – CEA	Indirect	Contingent valuation	Macroeconomic methods Econometric	Combined micro- & macro CGE models
Scope of evaluation	Technical efficiency of alternative health care interventions	Societal loss associated with disease prevention	Societal preferences for disease prevention and the relative importance of its attributes	Statistical association of communicable disease epidemiology with GDP or GDP per capita	Cross-sectorial microeconomic and macroeconomic impact of disease prevention
Evidence and analysis needs	Decision analytic modeling combining epidemiological data, resource use and unit costs; absenteeism, and presenteeism (optional)	Cohort modeling of epidemiological data, wages, absenteeism, and presenteeism	Survey collection and analysis methods	Econometric models to model retrospective panel data for GDP, epidemiology and a set of control variables	Sectorial matrix CGE models to simulate the consequence of disease on economic behaviors and productivity
Vaccinations’ benefit	Reduction of mortality and morbidity; Improvement of Patient quality of life; Prevention of health care costs; productivity gains and care-giving needs reduction	Reduction of sick-days; Increase of productivity while at work; Increase in total productive life years; increase in education levels achieved and lifetime education-specific earnings	Vaccinations are associated with positive WTP for preventing disease	Vaccination benefit can be quantified if vaccination variables are included in the model and vaccination data available	Change of economic behavior patterns projected/ investment choices
Decision criterion	ICERs in terms of cost/LY or QALY or DALY gained or cases averted	Inclusion in CEA or CBA	NPV: WTP for prevention of disease minus the cost of vaccination	Statistical relationship between GDP and vaccination	GDP impact of disease prevention; income, productivity gain, income distribution
Incorporation of broader consequences	Optional inclusion of absenteeism and presenteeism evaluations for the individual and the firm	Absenteeism and presenteeism evaluations for the individual and the firm; Quantification of the statistical and fiscal value of future cohorts (demographical changes); May link education outcomes with lifetime earnings	Depending on the survey design it may assess intangible elements that influence individuals’ decisions	Retrospective analysis implicitly captures all levels	Projections of the macroeconomic consequences are based on assumptions or data relating to broader consequences
Policy relevance/utility	Efficient allocation of resources within the health care budget	Comprehensive estimate of the economic surplus produced for the society	Identify societally preferred health policies	Ad-hoc assessment of cross – country macroeconomic association between the disease and the GDP; Cross-country best practice identifications	Cross-sectorial allocation of funding; Public investment appraisals
